# Koexistente septische Arthritis und Spondylodiszitis als wichtige Differenzialdiagnose bei immunsupprimierten Patienten

**DOI:** 10.1007/s00393-020-00943-8

**Published:** 2020-12-17

**Authors:** S. Pfahler, R. Pflugmacher, P. Karakostas, D. Dabir, V. S. Schäfer

**Affiliations:** 1 Medizinische Klinik III, Onkologie, Hämatologie, Rheumatologie und klinische Immunologie, Universitätsklinikum Bonn, Venusberg Campus 1, 53127 Bonn, Deutschland; 2 Klinik und Poliklinik für Orthopädie und Unfallchirurgie, Universitätsklinikum Bonn, Bonn, Deutschland; 3 Klinik für Diagnostische und Interventionelle Radiologie, Universitätsklinikum Bonn, Bonn, Deutschland

**Keywords:** Septische Arthritis, Spondylodiszitis, Differentialdiagnose, Arthritis, Immunsuppression, Septic arthritis, Spondylodiscitis, Differential diagnosis, Arthritis, Immunosuppression

## Abstract

Die septische Arthritis und Spondylodiszitis stellen bei immunsupprimierten Patienten eine wichtige Differenzialdiagnose des Gelenk- oder Wirbelsäulenschmerzes dar. Hierbei kommt es zu einem Erregerbefall eines Gelenks bzw. einer Bandscheibe und angrenzender Wirbelkörper. Es zeigen sich meist unspezifische Symptome wie lokaler Gelenk- oder Rückenschmerz, Fieber und verringerter Allgemeinzustand. Diagnostisch kann bei klinischem Verdacht die bakterielle Besiedelung durch eine Gelenkpunktion und Blutkulturen nachgewiesen werden. Zur Diagnosefindung einer Spondylodiszitis sollte eine bildmorphologische Darstellung mittels Magnetresonanztomographie erfolgen. Neben einer adäquaten Schmerztherapie und empirischer antibiotischer Therapie sollte bei einer septischen Arthritis die chirurgische Entfernung des infektiösen Materials aus dem Gelenk angestrebt werden. Eine chirurgische Versorgung der Spondylodiszitis sollte bei auftretenden Komplikationen erfolgen. Die folgende Kasuistik stellt den gleichzeitigen Befund einer septischen Polyarthritis und Spondylodiszitis bei einem immunsupprimierten Patienten mit HIV-Infektion vor und zeigt eindrücklich das Auftreten von Komplikationen bei Verzögerung einer adäquaten Therapie.

## Abstract (en)

Septic arthritis and spondylodiscitis, especially in immunocompromised patients, constitute a major differential diagnosis of joint or back pain. This results in the invasion of a joint or spinal disc and its adjacent vertebral body by a pathogen. In most cases this is manifested as unspecific symptoms, such as local joint or back pain, fever and malaise. If this is clinically suspected the bacterial infection of the joint can be confirmed by joint puncture and blood culture. For the diagnosis of spondylodiscitis magnetic resonance imaging should be used for visualization. In addition to adequate pain treatment and empirical antibiotic treatment, an arthroscopic removal of infected intra-articular tissue and debris is imperative. When complications caused by spondylodiscitis arise a surgical removal and stabilization should be performed. The following case report presents the findings of septic polyarthritis and spondylodiscitis in an immunocompromised patient with an HIV infection and provides insights into the occurrence of complications due to the delay of adequate treatment.

## Einleitung

Die septische Arthritis ist eine wichtige Differenzialdiagnose bei immunsupprimierten Patienten. Auch ohne ersichtlichen Infektionsfokus oder Hergang sollte ein möglicher Erregerbefall im Zweifel durch eine Gelenkpunktion abgeklärt werden. Die Diagnose erfolgt aus dem Gelenkpunktat mit einer stark erhöhten Zellzahl (regulär >50.000 Zellen) sowie dem mikrobiologischen Erregernachweis. Die Verlaufskontrolle erfolgt klinisch sowie laborchemisch. Die Therapie stützt sich auf 2 Säulen: die angepasste antibiotische Therapie und eine Drainage oder – bei implantierten Materialien – eine offene chirurgische Entfernung des infektiösen Materials aus dem Gelenk.

## Fallbeschreibung

### Anamnese

Es wird über einen 46-jährigen Patienten berichtet, der sich in unserer rheumatologischen Ambulanz mit Polyarthralgien vorstellte. Der Patient berichtete von seit 4 Tagen bestehenden, starken Schmerzen (7/10) diffuser Natur, welche in der rechten Schulter, dem linken Knie sowie der Hals- und Lendenwirbelsäule bestünden. Ein Trauma oder eine Verletzung jeglicher Art wurden als Auslöser verneint. Seit 2 Tagen zeige sich jedoch eine Verschlechterung des Allgemeinzustands mit Fieber bis 39 °C. Stuhlgang und Miktion wären unauffällig. Zu nennende relevante Nebendiagnose ist eine HIV-Infektion (ED: 01/07), welche zuvor antiviral mit 1‑mal täglich Bictegravir 50 mg, Emtricitabin 200 mg und Tenofovir 25 mg behandelt wurde. Des Weiteren liegen eine chronische Hepatitis B mit Nachweis von HBV-DNA mittels PCR sowie eine Hepatitis-C-Infektion (Genotyp 1b) vor. Die HIV-Basistherapie wurde nach Angabe des Patienten selbstständig abgesetzt, wodurch ein Abfall der CD4^+^-Helferzellen von während der Therapie 1200/µl auf 635/µl zu messen ist. Es erfolgte keine gesonderte Therapie der Hepatitiden. Bei Zustand nach i.v.-Drogenabusus befindet sich der Patient in einer Substitutionstherapie mit L‑Polamidon. Der Patient berichtet von einer Penicillin-Allergie.

### Klinischer Befund

Klinisch zeigte sich ein kachektischer Patient in deutlich reduziertem Allgemeinzustand. Es ergaben sich keine Anzeichen auf eine kardiale oder respiratorische Problematik. Die Körpertemperatur wurde bei 38,3 °C im Ohr gemessen. Bei der klinischen Untersuchung zeigte sich eine deutliche Schwellung und Rötung des linken Kniegelenkes mit begleitendem Erguss sowie massivsten Schmerzen bei Bewegung. Die rechte Schulter wies ein positives Kapselmuster (schmerzhafte Außenrotation und Abduktion) auf. Weiterhin stellten sich druckschmerzhafte Schwellungen der Handgelenke dar. Ein eigenständiger Gang war schmerzbedingt nicht mehr möglich, weshalb der Patient mit einem Rollstuhl bewegt wurde. Ruhiges, aufrechtes Sitzen wurde durch Schmerzen der Hals- und Lendenwirbelsäule nur kurzzeitig toleriert. Auffällig war ein ausgeprägter Wirbelsäulenklopfschmerz über der gesamten Wirbelsäule.

### Labor

Laborchemisch waren bei Aufnahme ein erhöhtes CRP von 172 mg/l sowie im Blutbild normwertige Leukozyten mit 7,8 G/l und eine Neutrophilie mit 85,6 % ersichtlich. Die Bestimmung der HI-Viruslast im Blut ergab eine nachweisbare HIV-RNA von 1278 Kopien/ml. Mittels PCR konnte ein Nachweis von HBV-DNA mit <30 iU/ml erbracht werden. Eine Bestimmung der HCV-RNA zeigte 1,9 Mio. iU/ml.

### Therapie und Verlauf

**Figure 1:**
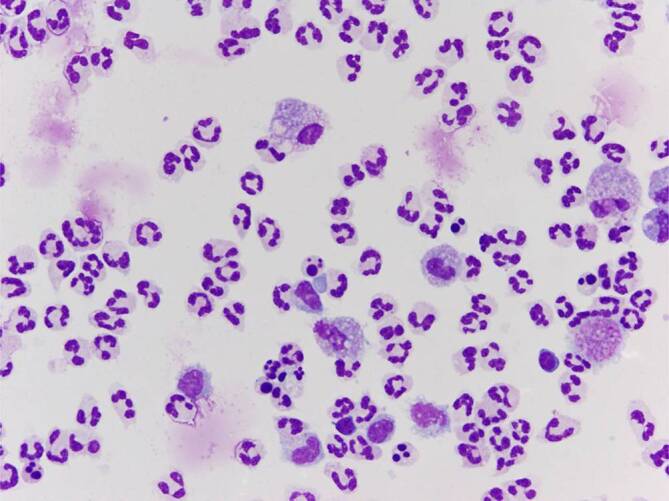

Aufgrund der massiven entzündlichen Kniegelenkschwellung wurde eine Kniegelenkpunktion durchgeführt. Diese ergab 70 ml eitrig, trübes Punktat (Abb. 1) mit 110.000 Leukozyten/µl. Mikroskopisch imponierten hauptsächlich stabkernige neutrophile Granulozyten. Die Gram-färbung des Punktats ergab den Nachweis von mäßig vielen grampositiven Haufenkokken. Eine anschließende kulturelle Auswertung zeigte im Verlauf *Staphylococcus aureus* ohne Resistenzen gegen Penicillin G, Cephalosporine, Vancomycin oder Clindamycin. Nach Sicherung der bakteriellen Arthritis durch Punktion des Kniegelenkes wurde eine stationäre Aufnahme durch die orthopädischen Kollegen der Universitätsklinik Bonn zur Einleitung einer empirischen antibiotischen Therapie veranlasst. Diese erfolgte mit 1,5 g Vancomycin i.v. 1‑0‑1 in Kombination mit 5 g Fosfomycin i.v. 1‑0‑1. Vor Beginn der antibiotischen Therapie wurden periphere Blutkulturen gewonnen, in welchen ebenfalls *Staphylococcus aureus* anzüchtbar war. Nach Abwägung wurde bei bestehender Penicillin-Allergie und besonderem Ausmaß der Erkrankung die antibiotische Therapie beibehalten. Es erfolgte eine Immobilisation des Kniegelenkes. Eine arthroskopische Spülung wurde durch den Patienten bei allgemeiner Skepsis gegenüber der ärztlichen Behandlung abgelehnt. Von einer zusätzlichen diagnostischen Punktion des Schultergelenks und der Handgelenke wurde bei geringen Entzündungszeichen und nicht punktionswürdigem Erguss der Gelenke abgesehen.

### Mikroskopisches Bild des Gelenkpunktats

Aufgrund massiv ansteigender Schmerzsymptomatik im Bereich der Halswirbelsäule wurde notfallmäßig zudem eine holospinale MR-Diagnostik durchgeführt. In dieser stellte sich eine Spondylitis der HWK 4–7 mit paravertebralem und epiduralem Abszess dar (Abb. 1). Hinweise auf eine Myelopathie konnten nicht erbracht werden. Trotz ausführlicher Aufklärung der möglichen Komplikationen bei Verzicht auf eine Operation wurde durch den Patienten die Weiterführung der konservativen Therapie mittels i.v.-Antibiose und i.v.-Analgesie ohne jegliche invasive Maßnahmen gewünscht. Unter dieser Therapie zeigte sich ein langsamer Rückgang der Beschwerdesymptomatik mit Verbesserung der Entzündungsparameter (CRP: 82 mg/l; Leukozyten 13 G/l).

**Figure 2:**
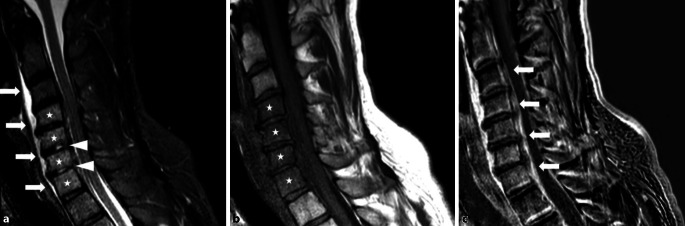

Nach 4 Tagen antibiotischer Therapie klagte der Patient über ein zunehmendes Schwächegefühl des linken Armes. Klinisch zeigte sich ein beginnendes Kraftdefizit des linken Triceps brachii (4/5). Im linken Kniegelenk waren weiterhin eine größenkonstante Schwellung und Erwärmung tastbar. Eine erneut angefertigte MR-Untersuchung der Halswirbelsäule zeigte eine Zunahme des intraspinalen Empyems, insbesondere in Höhe C5/6 und C6/7, sowie eine beginnende Spondylodiszitis (Abb. 2). Nach erneuter Aufklärung über die Dringlichkeit einer Infektsanierung stimmte der Patient einem operativen Vorgehen zu. Folglich wurden zur Infektausräumung eine Arthroskopie des linken Kniegelenks sowie eine ventrale Spondylodese C5–C7 mit Nukleotomie C5/6 und C6/7 und anschließender Cage-Interposition durchgeführt. Hierbei gewonnene Proben des Sekretes der Bandscheibenfächer zeigten in der kulturellen Untersuchung mäßig viel *Staphylococcus aureus*.

### MRT-Aufnahmen der HWS

Im postoperativen Verlauf blieb die oben geschilderte neurologische Ausfallsymptomatik unverändert, weshalb eine MR-Kontrolluntersuchung durchgeführt wurde. In dieser zeigten sich Myelopathiezeichen auf Höhe von HWK 5 bei hochgradiger spinaler Enge. Trotz Indikation einer operativen Revision wurde diese durch den Patienten abgelehnt. Die kulturelle Untersuchung von peripher und zentral gewonnenen Blutkulturen zeigte 5 Tage postoperativ kein Wachstum. Nach Besserung der Entzündungsparameter auf ein CRP von 15 mg/l und Leukozyten 3,5 G/µl wurde der Patient in eine neurologische Anschlussheilbehandlung übergeben. Die antibiotische Therapie wurde 27 Tage postoperativ auf eine orale Gabe von 500 mg Levofloxacin 1‑0‑1 für weitere 8 Wochen umgestellt. Der Patient war im gesamten Behandlungsverlauf weitestgehend unkooperativ, weshalb konsiliarische Mitbeurteilungen durch kardiologische oder neurologische Kollegen generell nicht möglich waren.

## Zusammenfassung

Die septische Arthritis ist eine seltene, ernst zu nehmende Erkrankung, bei der es zu einem Erregerbefall von einem oder mehreren Gelenken kommt. Ursächlich können Bakterien wie im hier beschriebenen Fall ohne ursprünglich traumatische Eröffnung eines Gelenkes, über hämatogene Streuung oder Ausbreitung per continuitatem in die Gelenkregion gelangen [5]. *Staphylococcus aureus* stellt sich hierbei als häufigster Erreger dar (Tab. 1; [10]). Gerade Patienten mit im Rahmen einer HIV-Infektion bestehender Immunsuppression haben eine erhöhte Inzidenz einer septischen Arthritis. Das klinische Bild zeigt typischerweise eine Schwellung, Rötung und Überwärmung der betroffenen Gelenke. Dies kann mit Schmerzen und Bewegungseinschränkungen einhergehen. Häufig entwickeln die Patienten Fieber [4]. Als typischer Manifestationsort der Infektion stellt sich wie bei dem beschriebenen Patienten das Kniegelenk heraus. Zur Diagnostik der septischen Arthritis sollten neben einer gezielten Anamnese und ausführlicher körperlicher Untersuchung die Abnahme von mindestens 2 Blutkulturen sowie eine Punktion des betroffenen Gelenks mit Gewinnung von Gelenkflüssigkeit erfolgen. Diese sollten umgehend der Bestimmung der Zellzahl sowie der Gram-Färbung und ggf. indizierter mikrobiologischer Diagnostik zugeführt werden. Laborparameter wie BSG, CRP und Leukozyten haben nur eine bedingte diagnostische Relevanz, sollten jedoch zur Verlaufskontrolle herangezogen werden [5]. Weiterhin ist eine radiologische Bildgebung in 2 Ebenen von den betroffenen Gelenken durchzuführen, um Zeichen einer inflammatorischen Gelenkveränderung wie erosive oder osteolytische Vorgänge zu erfassen. Als schnell verfügbare erweiternd bildgebende Maßnahme kann mittels Gelenksonographie der betroffenen Gelenke eine zusätzliche Aussage über das Vorliegen einer Synovitis erfolgen [7]. Nach Sicherung der Diagnose muss unverzüglich mit der Einleitung einer empirischen antibiotischen Therapie begonnen werden. In der aktuellen S1-Leitlinie werden Gruppe-2-Cephalosporine empfohlen [2]. Unter Berücksichtigung der individuellen Krankengeschichte wurde in diesem Fall wegen Penicillin-Allergie eine Therapie mit Vancomycin und Fosfomycin i.v. gewählt. Initial sollte eine intravenöse Gabe des Antibiotikums stattfinden, um einen ausreichenden Wirkspiegel im Gelenk zu erzielen [8]. Die Therapie sollte ggf. auf den mikrobiologischen Befund mit Resistenztestung angepasst werden. Neben der antibiotischen Therapie sollte eine chirurgische Intervention zur Reduktion der Keimzahl erfolgen. Grundsätzlich mögliche Verfahren sind eine Punktion mit Einlage von Drainagesystemen, die Spülung durch Arthroskopie oder eine offene Dekompression mittels Arthrotomie [7]. Aufgrund mangelnder Compliance des Patienten erfolgte ein später chirurgischer Eingriff, wodurch eine deutliche Verschlechterung der Symptomatik auftrat. Grundsätzlich sollten eine ausreichende Analgesie und frühfunktionelle Bewegungstherapie durch Physiotherapeuten erfolgen [5].

**Table 1:** 

Häufige Erreger einer septischen Arthritis [7]	Häufige Erreger einer Spondylodiszitis [9]
*Staphylococcus aureus* (56 %)	*Staphylococcus aureus* (20–84 %)
Streptokokken (16 %)	Gramnegative Erreger (7–33 %)
Gramnegative Erreger (15 %)	Enterokokken und Streptokokken (5–20 %)
Andere (12 %)	Koagulase-negative Staphylokokken (5–16 %)

Die in diesem Fall begleitende Spondylodiszitis beschreibt die infektionsbedingte Entzündung der Bandscheiben und angrenzender Wirbelkörper. In Europa stellt auch hier *Staphylococcus aureus*, gefolgt von *E. coli* [5], den häufigsten Erreger dar (Tab. 1; [5]). Häufigster Infektionsmechanismus ist hierbei die hämatogene Streuung [5], in diesem Fall wahrscheinlich ausgehend von der zuvor beschriebenen septischen Arthritis. Immunsuppression z. B. durch eine HIV-Infektion, intravenöser Drogenkonsum und Alkoholabusus führen zu einem erhöhten Risiko, das Krankheitsbild zu entwickeln [3]. Wie im Fallbeispiel manifestiert sich die Spondylodiszitis meistens durch unspezifische Rückenschmerzen im betroffenen Bereich. Fieber und eine Reduktion des Allgemeinbefindens können als begleitendes Symptom der Infektion auftreten. Werden durch die Infektion die Wirbelkörper oder benachbarte Strukturen destruiert, können entsprechende Symptome auftreten. Gefürchtete Komplikationen stellen paravertebrale Abszesse und – wie im Fallbeispiel geschildert – neurologische Ausfallerscheinungen dar [5]. Eine rasche Erregeridentifikation mittels Blutkultur oder CT-gesteuerter Punktion [6] und die Bestimmung von Laborparametern als Verlaufsparameter des Entzündungsgeschehens [5] werden analog zur septischen Arthritis empfohlen. Unklare Symptome der Wirbelsäule sollten initial mittels Röntgenbildes des entsprechenden Wirbelsäulenabschnitts abgeklärt werden. Als diagnostisches Verfahren stellt – wie in Abb. 2 zu sehen – die kontrastmittelgestützte MRT den Goldstandard dar. Bei bestehenden Kontraindikationen zur MRT kann eine CT als alternatives diagnostisches Verfahren eingesetzt werden [5]. Schmerzlinderung, Funktionserhalt und -wiederherstellung des betroffenen Segments sowie Beseitigung der bakteriellen Infektion stellen Hauptziele der Therapie einer Spondylodiszitis dar. Bei Patienten ohne neurologische Defizite und mit stabilen hämodynamischen Bedingungen sollte eine empirische Antibiose erst nach Identifikation des Erregers erfolgen [1]. Eine initiale Therapie sollte zur ausreichenden Bioverfügbarkeit intravenös erfolgen. Anschließend kann auf orale Antibiotika mit guter Bioverfügbarkeit und Gewebegängigkeit umgestellt werden. In diesem Fall wurde Levofloxacin 500 mg 1‑0‑1 gewählt. Durch das Auftreten einer Komplikation mit neurologischem Defizit, Sepsis und intraspinalem Empyem bei Versagen der konservativen Therapie wurde leitliniengerecht eine chirurgische Intervention mit Beseitigung des Infektfokus und Stabilisierung des Wirbelsäulensegments angestrebt [5].

## Fazit für die Praxis

- In der Differenzialdiagnose von Polyarthralgien sollte insbesondere bei weiteren Hinweisen auch eine septische Arthritis erwogen werden.
- Eine ätiologisch unklare Monoarthritis sollte einer Punktion zugeführt werden.
- *Staphylococcus aureus* ist gefolgt von Streptokokken und *Escherichia coli* der häufigste Erreger einer septischen Arthritis.
- Patienten mit einer septischen Arthritis sollten umgehend antibiotisch behandelt und zeitnah einer arthroskopischen Gelenkspülung unterzogen werden.
- Bei Rückenschmerzen als Begleitsymptom einer septischen Arthritis sollte ohne Verzug eine Spondylodiszitis mittels Bildgebung (MRT) weiter abgeklärt werden.
